# Zika vector competence data reveals risks of outbreaks: the contribution of the European ZIKAlliance project

**DOI:** 10.1038/s41467-022-32234-y

**Published:** 2022-08-02

**Authors:** Thomas Obadia, Gladys Gutierrez-Bugallo, Veasna Duong, Ana I. Nuñez, Rosilainy S. Fernandes, Basile Kamgang, Liza Hery, Yann Gomard, Sandra R. Abbo, Davy Jiolle, Uros Glavinic, Myrielle Dupont-Rouzeyrol, Célestine M. Atyame, Nicolas Pocquet, Sébastien Boyer, Catherine Dauga, Marie Vazeille, André Yébakima, Michael T. White, Constantianus J. M. Koenraadt, Patrick Mavingui, Anubis Vega-Rua, Eva Veronesi, Gorben P. Pijlman, Christophe Paupy, Núria Busquets, Ricardo Lourenço-de-Oliveira, Xavier De Lamballerie, Anna-Bella Failloux

**Affiliations:** 1 Institut Pasteur, Université Paris Cité, Bioinformatics and Biostatistics Hub, F-75015 Paris, France; 2 Institut Pasteur, Université Paris Cité, G5 Infectious Disease Epidemiology and Analytics, F-75015 Paris, France; 3 Department of Vector Control, Center for Research, Diagnostic, and Reference, Institute of Tropical Medicine Pedro Kouri, Havana, Cuba; 4 Institut Pasteur of Guadeloupe, Laboratory of Vector Control Research, Unit Transmission Reservoir and Pathogens Diversity, Les Abymes, Guadeloupe; 5 Institut Pasteur du Cambodge, Virology Unit, Phnom Penh, Cambodia; 6 IRTA, Centre de Recerca en Sanitat Animal (CReSA, IRTA-UAB), Campus de la Universitat Autònoma de Barcelona, 08193 Bellaterra, Spain; 7 Laboratorio de Mosquitos Transmissores de Hematozoarios, Instituto Oswaldo Cruz, Fiocruz, Rio de Janeiro, RJ Brazil; 8 Centre for Research in Infectious Diseases, Department of Medical Entomology, Yaoundé, Cameroon; 9 UMR PIMIT (Processus Infectieux en Milieu Insulaire Tropical), Sainte-Clotilde, La Réunion, France; 10 Laboratory of Virology, Wageningen University, Wageningen, The Netherlands; 11 IRD, MIVEGEC, University of Montpellier, IRD, CNRS, Montpellier, France; 12 National Centre for Vector Entomology, Institute of Parasitology, Vetsuisse Faculty, University of Zürich, Zürich, Switzerland; 13 Institut Pasteur de Nouvelle-Calédonie, URE Dengue et Arboviroses, Nouméa, New Caledonia; 14 Institut Pasteur de Nouvelle-Calédonie, URE Entomologie Médicale, Nouméa, New Caledonia; 15 Institut Pasteur du Cambodge, Medical Entomology Unit, Phnom Penh, Cambodia; 16 Institut Pasteur, Université Paris Cité, Arboviruses and Insect Vectors, F-75015 Paris, France; 17 VECCOTRA, Rivière Salée, Martinique; 18 Laboratory of Entomology, Wageningen University & Research, Wageningen, The Netherlands; 19 Unité des Virus Emergents (UVE), Aix Marseille Université, IHU Méditerranée Infection, Marseille, France

**Keywords:** Virus-host interactions, Entomology

## Abstract

First identified in 1947, Zika virus took roughly 70 years to cause a pandemic unusually associated with virus-induced brain damage in newborns. Zika virus is transmitted by mosquitoes, mainly *Aedes aegypti*, and secondarily, *Aedes albopictus*, both colonizing a large strip encompassing tropical and temperate regions. As part of the international project ZIKAlliance initiated in 2016, 50 mosquito populations from six species collected in 12 countries were experimentally infected with different Zika viruses. Here, we show that *Ae. aegypti* is mainly responsible for Zika virus transmission having the highest susceptibility to viral infections. Other species play a secondary role in transmission while *Culex* mosquitoes are largely non-susceptible. Zika strain is expected to significantly modulate transmission efficiency with African strains being more likely to cause an outbreak. As the distribution of *Ae. aegypti* will doubtless expand with climate change and without new marketed vaccines, all the ingredients are in place to relive a new pandemic of Zika.

## Abstract (web summary)

Zika virus (ZIKV), the causative agent of virus-induced brain damage in newborns, is transmitted by mosquitoes, mainly *Aedes aegypti*, and secondarily, *Aedes albopictus*. Here, Obadia et al. characterize ZIKV vector competence of 50 mosquito populations from six species collected in 12 different countries to inform about epidemic risk. They find that African ZIKV strain shows higher transmission efficiency compared to American and Asian ZIKV strains and that *Ae. aegypti* mosquitoes have highest susceptibility to infections, while *Culex*mosquitoes are largely non-susceptible.

## Introduction

Viral pathogens with high epidemic potential have marked human history by massive losses of life and economic declines. Pandemics like the Spanish flu^1^ have left traces and the fear of new viral emergences materializes today with the Severe Acute Respiratory Syndrome-Coronavirus-2 (SARS-CoV-2) pandemic. Over the past decades, efforts invested into vaccination programs and development of antiviral treatments have led to major medical progress resulting in measles decline, smallpox eradication, appropriate treatments of Human Immunodeficiency virus and Hepatitis C virus, and improved control of SARS-CoV-2. As major viral pathogens, arthropod-borne viruses (arboviruses) are also on the rise. Arboviruses have developed a strategy to accomplish their transmission cycle *via* replication in both vertebrate and arthropod hosts. Their RNA genome contributes to the generation of large populations of genetically distinct variants permitting adaptations to environmental changes including host switching and transmission^2^. In the last 30 years, mosquito-borne viruses have dramatically expanded their distribution range leading to increasingly frequent and large epidemics^3^. In 2016, Zika was designated as a “Public Health Emergency of International Concern” by the World Health Organization, subsequently to the large spread of Zika virus (ZIKV) in the Pacific islands and the Americas with unusual notifications of microcephaly in newborns and different neurological disorders (e.g. Guillain-Barré syndrome); this happened 70 years after its first isolation from a human case in East Africa^4^. Local cases of Zika were detected in 2019 in Southern France additionally corroborating the role of the Asian tiger mosquito (*Aedes albopictus*) in ZIKV transmission in addition to chikungunya and dengue viruses^5^.

*Aedes* mosquitoes are important vectors of arboviruses, acquiring the virus through a blood meal from an infected host. Subsequently, the virus replicates within the mosquito and is transmitted to a new host. The virus must overcome two anatomical barriers (midgut and salivary glands) and circumvent major antiviral pathways before being delivered in saliva^6^. Geographic populations of a same mosquito species may not share the same immunological background, leading to different vector competences^7^. The outcome of infection depends on specific pairings of mosquito and pathogen genotypes described under genotype-by-genotype (G x G) interactions^8^. These interactions could structure viral populations through adaptations to their local vector populations^8^. Vector competence data have been used to derive risk maps informed with distribution of mosquito species, to help focus and strengthen vector control measures^9^. Nevertheless, other factors, non-genetic, biotic and abiotic, should be considered in the functioning of the vectorial system making it even more complex than initially thought^10^. A global vector competence analysis of potential Zika vectors (*Aedes aegypti*, *Ae. albopictus, Aedes japonicus, Culex pipiens pipiens, Culex pipiens molestus*, and *Culex quinquefasciatus)* was performed to assess the risk of Zika disease outbreaks. Once the vector is well established in a region, associated arboviruses have the potential to emerge through imported human cases. To this aim, we analyzed the vector competence of 50 mosquito populations from 15 locations and 12 countries (Brazil, Cambodia, Cameroon, Congo, Cuba, France, Gabon, Guadeloupe, Haiti, La Reunion, Madeira, The Netherlands, New Caledonia, Spain, and Switzerland) which were challenged with different ZIKV genotypes (West Africa, Asia, America) using standardized experimental protocols (mosquito infections, viral titrations, parameters of vector competence). In this work, we elaborated a vector competence data-driven prediction for ZIKV transmission to inform on the current risk of Zika transmission at a global scale. We show that *Ae. aegypti* populations tested are highly competent to transmit the different ZIKV genotypes and more specifically, the African genotype. Moreover, other mosquito species such as *Ae. albopictus* or *Ae. japonicus* can play a secondary role in transmission while *Culex* mosquitoes are definitively non-susceptible to ZIKV.

## Results

*Culex* spp. mosquitoes were resistant to most ZIKV strains, successfully blocking infection with an overall infection rate (IR) of 0.2% (2 mosquitoes out of 1089 tested) and complete absence of both dissemination efficiency (DE) and transmission efficiency (TE). As such, they were not included in more complex vector competence analyses. *Aedes* spp. showed different patterns depending on species and ZIKV strain used for infection. For *Ae. aegypti* and *Ae. albopictus*, the experimental design required nested, within-country random effects. In the case of *Ae. japonicus*, the country and mosquito population effects were fully colinear, therefore analyses only relied on logistic regressions.

### Differential susceptibility of *Aedes* spp. mosquitoes to ZIKV

**Fig. 1:**
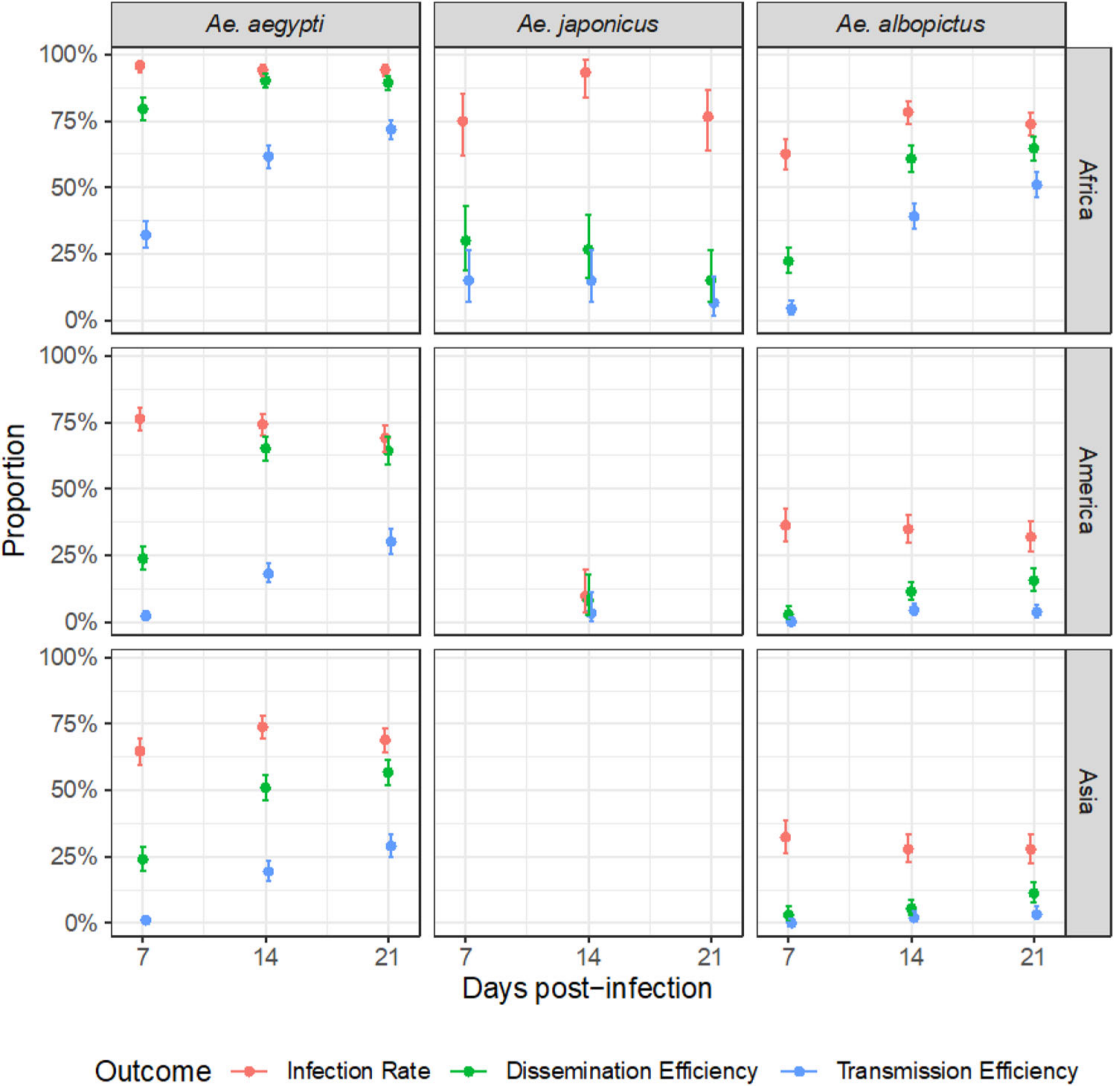
Infection rate, dissemination and transmission efficiencies of ZIKV strains (Africa, America, Asia) according to time since infection in three *Aedes* mosquito species. Panels show the rates achieved by averaging over all sampled mosquitoes, i.e., by pooling together mosquitoes from all countries used in the study. Error bars are exact 95% binomial confidence intervals centered on the observed means of IR, DE and TE. (*n* = 30 biologically independent mosquitoes for each combination of species, country, mosquito population, days post-infection and ZIKV strain were studied, unless stated otherwise. See Supplementary Table 1 for complete break-down of sample sizes). Colors correspond to the studied outcome (IR, DE, TE).

Depending on mosquito species and ZIKV strain, we observed different patterns of viral progression within the vector’s organs. In *Ae. aegypti* infected with ZIKV strains, there were no notable differences between IR and DE at 21 days post-infection (dpi), with IR = 94.3% (95% CI: [92.1%–96.0%]) and 69.1% [64.0%–73.9%], and with DE = 89.4% [86.6%–91.8%] and 64.3% [59.1%–69.3%] for respectively, African and American strains. However, there was a large and significant decrease for TE = 71.9% [68.0%–75.6%] and 30.0% [25.3%–35.1%] for respectively, African and American strains as can be seen in Fig. 1. This is consistent with efficient viral progression through the midgut, and partial barriers to viral progression before reaching the salivary glands. In contrast, when infected with Asian ZIKV strains, both midguts and salivary glands mitigated progression of the virus: IR = 68.9% [64.3%–73.3%], DE = 56.7% [51.8%–61.5%], and TE = 28.9% [24.7%–33.5%].

In *Ae. albopictus* mosquitoes, viral progression was more efficient with the African ZIKV strain than with American or Asian ZIKV strains for all three outcomes. For the African ZIKV strain, a regular decrease was observed between IR (73.9%), DE (69.2%) and TE (55.8%), consistent with the absence of a marked barrier to viral progression through the midgut and into the salivary glands. In contrast, DE was significantly lower for American (15.6% [11.5–20.4%]) and Asian (11.2% [7.7–15.5%]) ZIKV strains, consistent with a lack of viral progression through the midgut.

Time since infection showed an increasing, approximately linear association with DE and TE in *Ae. aegypti* and *Ae. albopictus* mosquitoes, suggesting an accumulation pattern and an overall absence of viral clearance within the vector, while IR reached a peak value within 7 dpi and remained at comparable levels until 21 dpi. In contrast, *Ae. japonicus* infected by the African ZIKV strain showed that IR peaked at 14 dpi, with decreasing DE and TE values over time (TE_7dpi_ = 15.0% [7.1–26.6%], TE_14dpi_ = 15.0% [7.1–26.6%] and TE_21dpi_ = 6.7% [18.5–16.2%]).

### Transmission efficiency is higher with the African ZIKV

Although all species of *Aedes* studied were competent to transmit ZIKV, infected *Ae. aegypti* species were on average more prone to successful dissemination than *Ae. albopictus* and *Ae. japonicus* (TE_*aegypti*_ = 34.0% [24.0–45.8%], TE_*albopictus*_ = 17.6% [11.4–26.0%], TE_*japonicus*_ = 7.2% [2.9–16.9%]). This was even more pronounced at 21 dpi when TE was significantly higher for *Ae. aegypti* than *Ae. albopictus* (46.7% [35.3–58.5%] vs. 25.3% [17.3–35.5%], *p* < 0.0001) and *Ae. japonicus* (46.7% [35.3–58.5%] vs. 10.8% [4.4–24.1%], *p* = 0.0002), the latter two not reaching statistical significance (*p* = 0.09). However, many combinations of ZIKV strains and mosquito populations were not available for *Ae. japonicus* for which results should be considered less robust.

**Fig. 2:**
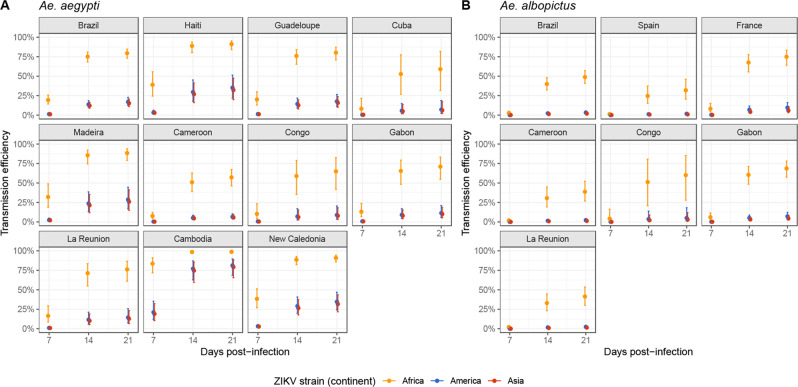
Model-predicted transmission efficiency of *Aedes* mosquitoes according to time since infection, split by country of mosquito sampling. TE predicted for (**A**) *Ae. aegypti* and (**B**) *Ae. albopictus*. Countries are ordered to reflect geographic proximity. Error bars show asymptotic 95% confidence interval from the mixed regression models centered on the average predictions of IR, DE and TE. (n = 30 biologically independent mosquitoes for each combination of species, country, mosquito population, days post-infection and ZIKV strain were studied, unless stated otherwise. See Supplementary Table 1 for complete break-down of sample sizes). Colors correspond to continent-aggregated ZIKV strains.

The African ZIKV strain was the most efficient in infecting and disseminating, therefore posing a greater risk in case it was newly introduced in a region: the predicted expected marginal means 21 dpi were TE = 82.7% [78.5–86.1%] for *Ae. aegypti* and TE = 52.4% [45.4–59.3%] for *Ae. albopictus* (Fig. 2). American and Asian strains had comparable TE regardless of time since infection: (1) in Cameroon, Congo, and Gabon, TE was low (~10%), suggesting African mosquitoes are mostly susceptible to local ZIKV strains; (2) in Brazil, Haiti, Guadeloupe, Madeira, La Reunion and New Caledonia, TE reached moderate values ~20%. In Cambodia however, Asian and American ZIKV strains had much higher TE values, denoting mosquito populations highly susceptible to ZIKV.

### Breaking down variable importance in ZIKV susceptibility

**Fig. 3:**
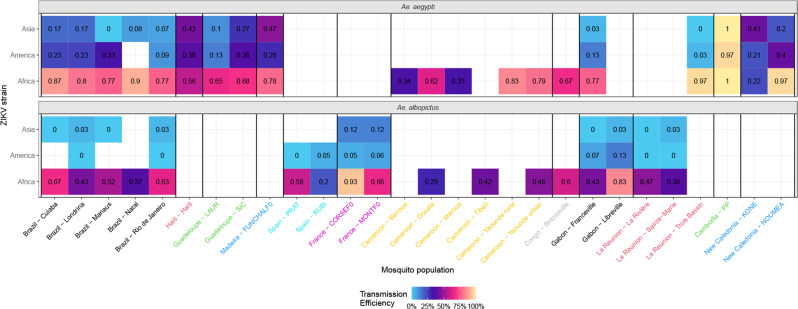
Transmission efficiencies of ZIKV strains at the regional level for *Ae. aegypti* and *Ae. albopictus* mosquitoes sampled at one or more locations in every studied country, at 21 dpi. ZIKV strains were pooled in a geographical clustering that reflected their phylogeny. Vertical bars help identify different countries, shown on the *x*-axis with individual colors. Empty cells represent absence of data. Color gradients correspond to TE values (ranging 0–100%).

We evaluated the extent to which each covariate of interest contributed to explanatory value of fitted models. For both *Ae. aegypti* and *Ae. albopictus*, TE showed low variability among mosquito populations originating from the same country (Fig. 3). Another striking result from Fig. 3 confirmed the higher susceptibility to African ZIKV strains among all tested mosquitoes, both within and across countries. This suggests that within a given country, mosquito populations were equally able to disseminate ZIKV regardless of other extrinsic factors. We found that 51.7% of variance in *Ae. aegypti* TE could be explained by a statistical model accounting for mosquito strain, ZIKV strain, and dpi (Table 1). The relative importance of these factors was assessed by estimating the proportion of variance explained solely by these factors. For *Ae. aegypti* TE, ZIKV strain was the most important factor explaining 33.5% of variance, followed by dpi (30.1%). Of the factors considered, mosquito strain had the least impact on TE, with only 6.6% of variance explained by the country of a sampled mosquito strain. An additional 2.6% of variance was explainable when we considered locations of strains within a country. For *Ae*. *japonicus*, no obvious pattern could be detected with only 5.2% of the observed variance explained by available covariates suggesting that mosquitoes were less susceptible to tested ZIKV strains.

**Table 1:** Contribution of random effects and fixed effects in species-wise regression models

	Total variance explained	Variance uniquely explained			
Mosquito species		Within-country (mosquito)	Between-country (mosquito)	ZIKV strain	Days post-infection
*Ae. aegypti*	51.7%	2.6%	6.6%	33.5%	30.1%
*Ae. japonicus*	5.2%		1.0%	0%	1.0%
*Ae. albopictus*	41.5%	1.7%	0%	29.0%	21.2%

The contribution of random effects was measured as the adjusted intra-class correlation coefficient (as defined in Nakagawa et al.^52^), while the contribution of fixed effects was assessed by the semi-partial *R*^2^ measure (from Stoffel et al.^51^), i.e. the share of variance uniquely explained by a given covariate. The total variance explained is the *R*^2^ measure of the saturated model.

## Discussion

Our results confirmed that human-adapted *Aedes* mosquitoes prepare the field for new arbovirus emergences; in addition to other factors, some combinations of vector-virus are more prone to cause outbreaks, but globalization will smooth out differences leading most emergences to turn into pandemics owing to the large territories covered by vectors.

During the 2015–2016 Latin American and the Caribbean epidemic, the role of *Culex* as ZIKV vector has been the subject of major scientific debates which, if verified, would have revised the *modus operandi* of vector control^11^. As ZIKV has also been detected in field-collected *Culex* mosquitoes^12,13^, their susceptibility to ZIKV was then tested; from 36 studies on vector competence of *Culex* mosquitoes for ZIKV, seven studies showed that ZIKV was present in the salivary glands of *Cx. restuans*, *Cx. quinquefasciatus*, *Cx. tarsalis*, and *Cx. Coronator*. Asian and American genotypes of ZIKV may occasionally infect *Culex* mosquitoes but not African strains^14^. From our dataset^15–18^, we showed that ZIKV can accumulate in *Cx. pipiens* saliva only after forced infection by intrathoracic inoculation, short-circuiting the midgut barrier^17^; after oral infection, the virus was unlikely to disseminate in the mosquito ruling out the role of *Culex* mosquitoes in ZIKV transmission. Viral replication was not detected in *Culex* which was unable to trigger the RNAi pathway as no viral replication signatures such as 21 nt viral siRNAs were detected^19^. Even though vectors of the flavivirus West-Nile virus, a phylogenetically ZIKV-related arbovirus, mosquitoes of the *Cx. pipiens* complex are not competent for ZIKV transmission and are very unlikely to play a role of vector during a Zika outbreak. Thus, to control Zika, targeted interventions should focus largely on *Aedes* mosquitoes^11,20^.

*Aedes aegypti* is an African mosquito (named *Ae. aegypti formosus*) living primarily in rainforests colonizing natural breeding sites which in a subsequent step, has become adapted to living in the human environment (developing a human-biting behavior and colonizing human-made containers) before undergoing different waves of dissemination outside Africa^21^. The “domestication” of this species (named *Ae. aegypti aegypti*) allowed its world-wide dispersion coinciding with the first twentieth century pandemics caused by arboviruses, dengue^22^, and then chikungunya^23^ and Zika^24^. In addition to increased vector-host contacts, *Ae. aegypti aegypti* has developed an enhanced permissiveness to some arboviruses^7^. At the mosquito level, successful transmission implies that the virus passes through two anatomical barriers, midgut and salivary glands^25^. *Ae. aegypti* mosquitoes we tested were more prone to impose a strong bottleneck to the African ZIKV in the salivary glands leading to decrease ZIKV transmission. Nevertheless, all 11 *Ae. aegypti* populations transmitted at least 2–3 times more the African ZIKV than the American and Asian ZIKV strains underlining the high potential of African ZIKV to trigger an outbreak outside and inside Africa. It is now admitted that African ZIKV strains were more pathogenic than the Asian strains^26,27^. African *Ae. aegypti* (Cameroon, Congo, Gabon) were more resistant to American and Asian ZIKV than to African ZIKV suggesting a local adaptation between a ZIKV strain and a mosquito population. There is evidence today that *Ae. aegypti formosus* is found both in urban and forest habitats in sub-Saharan Africa^28^ and a mixture of *Ae. aegypti aegypti* and *Ae. aegypti formosus* is likely present in cities we sampled in Cameroon, Congo and Gabon^29^. As *Ae. aegypti aegypti* is more susceptible to ZIKV than *Ae. aegypti formosus*, an intermediate level of susceptibility can be expected^7^. *Ae. aegypti formosus*, which is predominant in Benoué, was previously found less susceptible to ZIKV compared to the other populations from Cameroon^30^. Nevertheless, mosquitoes were only identified according to morphological criteria^31^ and not based on their genotypes. As such, the observed local reduction in vector competence may also result from other inherited factors not detected through morphological examinations. Our experiments showed a decreased susceptibility within the country, with *Ae. aegypti* from Benoué having lower TE (34.4% [20.2–52.1%]) than those sampled elsewhere in Cameroon (ranging from 62.5 to 83.3%, Supplementary Table 2) except in Maroua, located close to Benoué. Intriguingly, human epidemics of ZIKV associated with *Ae. aegypti* have been observed in a limited number of countries in Asia or Africa following the 2015–2016 Latin American and the Caribbean epidemic^24^. The Asian *Ae. aegypti* population from Cambodia we tested, transmitted the most efficiently all three ZIKV strains, African, American and Asian, corroborating its good vector competence to ZIKV. Recent systematic screenings of patients revealed that ZIKV was circulating in Singapore, Thailand, Cambodia, Myanmar and Vietnam suggesting that ZIKV has been present in Asia at a low but sustained level for years or even decades^32–35^. Moreover, human population immunity could also modulate the risk of outbreaks^36^; a large population exposed to dengue viruses may develop neutralizing antibodies against dengue viruses which cross-react with ZIKV adjusting the outcome of ZIKV infection^36^. Thereby, Asian populations can be partially protected against ZIKV^37^. At an intra-country level, mosquito populations (in our study, e.g. five in Brazil^18^) share the same profiles of transmission with a highest transmission level with African ZIKV. Once introduced, the African ZIKV has the potential to cause an outbreak whatever the origin of *Ae. aegypti* populations. Our assessments are based on vector competence which is only one aspect of vectorial capacity; it results from complex interactions of multiple factors influencing pathogen transmission by a vector, including internal factors such as genetic, evolution, immunity, or interactions with other microorganisms, modulated by external factors such as abiotic (like climate or topography) and biotic (like nutrition or hosts)^38^.

In the tropical belt*, Ae. aegypti* and *Ae. albopictus* share the same ecological niche^39^. *Ae. albopictus* mosquitoes we tested were more susceptible to African ZIKV suggesting that the species can contribute to an epidemic like it happened in Gabon in 2007^40^. A lower transmission of American and Asian ZIKV was presumably related to a strong mosquito midgut barrier^30^. Since its arrival in Central Africa in 2000, *Ae. albopictus* plays a major role in the transmission of different arboviruses^41^. *Ae. albopictus* tends to predominate over *Ae. aegypti* in habitats where both species are sympatric^42^ and will certainly promote arbovirus spillovers at the edge of their natural sylvatic cycle owing to its generalist behavior (biting both humans and animals, and living in rural areas)^41^. *Ae. albopictus* from Central Africa are likely originated from South America which themselves were introduced from North America in 1986^43^. European *Ae. albopictus* are presumably a genetic mixture of populations from United States and La Reunion^43^, sharing similar profiles of ZIKV transmission with mosquitoes from La Reunion^16,44,45^. American ZIKV predominant during the 2015–2016 epidemic, was efficiently transmitted by *Ae. albopictus*, in Europe^5^ and Brazil^46^. In the absence of *Ae. aegypti*, *Ae. albopictus* is more likely to trigger ZIKV outbreaks when ZIKV comes from Africa.

Another invasive species, *Ae. japonicus* also experimentally transmits the African ZIKV^47^. Native to East Asia, *Ae. japonicus* arrived in Europe in 2000^41^ and is established in 12 European countries up to now^48^. Its role in pathogen transmission is still unclear even if arboviral ZIKV RNAs have been detected in field-collected mosquitoes^49^. Its high ability to become infected, and to a lower extent, to ensure viral dissemination and transmission, concomitantly to its human-biting behavior gives *Ae. japonicus* the status of being a competent vector of ZIKV^47,50^. These findings are however subject to caution, as the experimental design may not allow for generalization of our observations: *Ae. japonicus* could only be sampled and bred in European countries (France (*n* = 90), Switzerland (*n* = 90), the Netherlands (*n* = 62)). To add to this limitation, only two ZIKV strains were tested against these mosquitoes and fully collinear with the country of mosquito sampling: French- and Swiss-sampled *Ae. japonicus* were infected with an African ZIKV strain, whereas mosquitoes from the Netherlands were infected with an American ZIKV strain (see data structure in Supplementary Fig. 1). Therefore, we cannot distinguish between mosquito susceptibility and ZIKV strain potency.

We conclude that from our comprehensive assessment of vector competence, invasive *Aedes* mosquitoes set the bed for pandemic arboviruses; in the absence of the historical vector of human arboviruses, *Ae. aegypti*, other species such as *Ae. albopictus* or *Ae. japonicus* can take over as ZIKV vectors and transmit even more efficiently when infected with the African ZIKV.

## Methods

### Experimental procedures

**Fig. 4:**
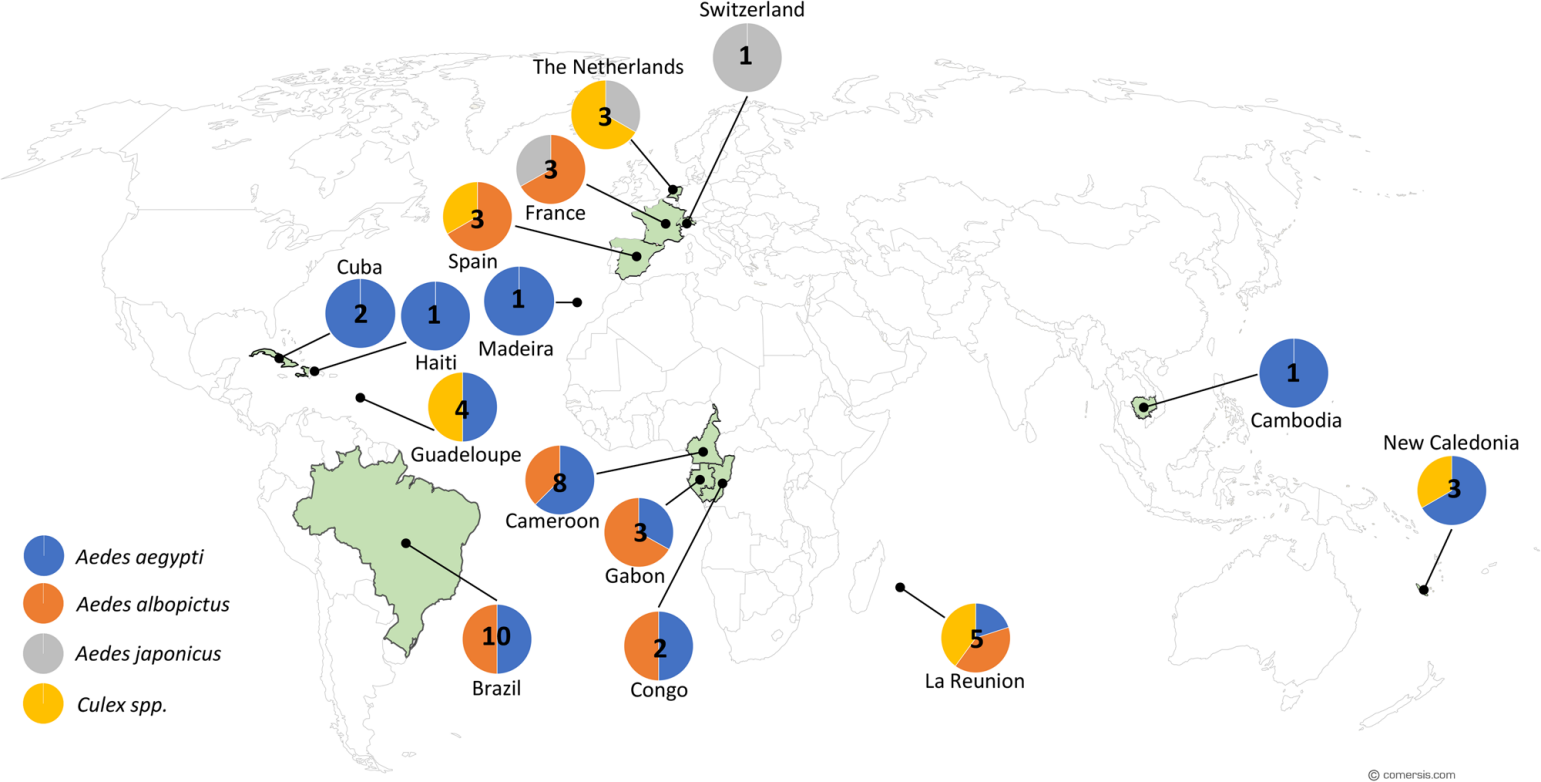
Distribution of mosquito species experimentally infected with Zika viruses. The color code used represents the different mosquito species and numbers refer to the number of mosquito populations tested. In green, are the countries sampled. Colors correspond to mosquito species. The map was built using the open source map site https://cmap.comersis.com/cartes-Monde-WORLD.html.

All partners of the ZIKAlliance project (https://zikalliance.tghn.org/) performed their own mosquito experimental infections using a common standardized protocol (Supplementary Fig. 2). Some deviations from this protocol could not be avoided (e.g. availability of blood source, feeding via blood droplets instead of *via* membrane). Fifty populations (Fig. 4 and Supplementary Table 1) were collected as immature stages and reared in insectaries under controlled conditions. Mosquito females were exposed to an infectious blood meal containing one third of viral suspension and two third of washed rabbit erythrocytes (Supplementary Fig. 3) at a final titer of ~10^7^ TCID_50_/ml. After 1 h, engorged females were isolated in boxes (28°±1 °C and 80 ± 10% humidity) and fed with 10% sucrose until analysis.

Different geographic ZIKV strains were used (Supplementary Fig. 4): (1) Africa (Dakar; KU955592; isolated in 1984 from *Aedes taylori* in Senegal and passaged four times on BHK21 cells), (2) Asia (Cambodia; KU955593; isolated from a human case in 2010 and passaged three times on Vero cells and Malaysia; KX694533; isolated in 1966 from *Aedes aegypti* in Malaysia and passaged four times on Vero cells), and (3) America (Guadeloupe; LR792671.1; isolated from a human case in Guadeloupe in 2016 and passaged two times on Vero cells, Martinique; KU647676; isolated from a human case in 2015 and passaged three times on Vero cells, and Suriname; KU937936; isolated from a human serum in Suriname in 2016 and passaged 5–6 times on Vero cells). ZIKV Dakar, Malaysia, and Martinique provided by EVAg (https://www.european-virus-archive.com/) were used in most infections. ZIKV Cambodia was used for the mosquito populations Haiti, Funchal, Mont, Corse, ZIKV Suriname for Best, Amsterdam and Lelystad, and ZIKV Guadeloupe for HAV-PT, HAV-PRG.

At 7, 14, and 21 days post-infection (dpi), a batch of mosquitoes was processed to estimate if viral particles were present in the body (thorax and abdomen) as the marker for infection, head for dissemination and saliva for transmission; 0 refers to no viral particles and 1 to at least one viral particle detected. Presence of viral particles was asserted by cytopathic effects observed on a monolayer of Vero CCL-81 cells in 96-well plates^45^. Three parameters were measured: (1) IR corresponding to the proportion of mosquitoes with infected body (thorax and abdomen) among examined mosquitoes, (2) DE referring to the proportion of mosquitoes with virus detected in the head among examined mosquitoes, and (3) TE representing the proportion of mosquitoes with virus detected in saliva among examined mosquitoes. TE was used as the primary endpoint to evaluate vector competence with respect to ZIKV strain. It is important to note that, by definition, TE ≤ DE ≤ IR, the denominator of each being the total number of mosquitoes dissected.

### Statistical analysis

Success for transmission, dissemination and infection were investigated as binomial outcomes to assess how their population-level counterpart (TE, DE, IR) were associated with available combinations of mosquito populations and ZIKV strains, according to time since infection. Time since infection was considered a categorical covariate. Prior to any modeling approach, all outcomes were visualized with heat maps similar to those presented in Supplementary Figs. 6, 8 and 9, for all combinations of ZIKV strain and mosquito population and at every value of time since infection. The resulting experimental design, consisting of combinations of country of study, mosquito species and populations as well as time since infection, was used to guide the development of generalized linear (mixed) models.

Owing to complex hierarchical experimental design, each mosquito species was modeled separately. When several mosquito populations were sampled at different locations within a given country, the variations resulting from the population effect was modeled by a random effect nested within the country, in a mixed regression framework. The contribution of each fixed-effect term was assessed by refitting univariable models and measuring the semi-partial R-squared value (as defined by Stoffel et al.^51^). Likewise, the contribution of random effects was measured as the adjusted intra-class correlation coefficient (as defined by Nakagawa et al.^52^). Analyses were conducted using the R software (v. 4.2.0)^53^ with the following packages: lme4 (v. 1.1-27.1)^54^, emmeans (v. 1.7.2), performance (v.0.8.0)^55^ and partR2 (v. 0.9.1).

The robustness of results was assessed using sensitivity analyses, where the same regression models were applied with data aggregated at larger geographical scales for covariates of interest. Three variations of the same analyses were conducted: (1) by aggregating country of study by continent (therefore assuming random variations across countries) and preserving individual ZIKV strains, (2) by aggregating ZIKV strains by continent (corresponding to relatedness between strains in the phylogenetic tree, see Supplementary Fig. 4) and preserving individual countries of study, and (3) aggregating both country and virus strain by continent. The results from aggregation detailed in point (2) are presented in the main text as they provide the most comprehensive set of results. The results corresponding to aggregation levels from points (1), (3) and (4) are presented in Supplementary Figs. 5 through 10.

### Reporting summary

Further information on research design is available in the Nature Research Reporting Summary linked to this article.

## Supplementary information

Supplementary Information

Reporting Summary

## Data Availability

The data that support the findings of this study are fully available in an electronic Supplementary table (Microsoft Excel XLSX format) at https://zenodo.org/record/6788898#.Ysgu0oTP2Uk as well as in the GitLab repository found in the Code availability section.

## Code availability

All analyses conducted in this manuscript and associated code have been uploaded to a Git repository at https://gitlab.pasteur.fr/tobadia/17289-zikalliance and are readily available. The results of the present manuscript are based on commit 1477a8be1554c91d2a5ea038964407e88499ddf9.
