# Supplementary material for: Zika vector competence data reveals risks of outbreaks: the contribution of the European ZIKAlliance project
Source: Nat Commun. 2022 Aug 2;13:4490. doi: 10.1038/s41467-022-32234-y (PMC9345287; doi:10.1038/s41467-022-32234-y)
Supplement: Supplementary file 1 — Supplementary Information [file 41467_2022_32234_MOESM1_ESM.docx]

**Supplementary information**

**Zika vector competence data reveals risks of outbreaks**

**The contribution of the European ZIKAlliance project**

**Thomas Obadia^1,17^, Gladys Gutierrez-Bugallo^2,7^, Veasna Duong^3^, Ana I. Nuñez^4^, Rosilainy S. Fernandes^5^, Basile Kamgang^6^, Liza Hery^7^, Yann Gomard^8^, Sandra R. Abbo^9^, Davy Jiolle^10^,** [**Uros Glavinic**](https://pubmed.ncbi.nlm.nih.gov/?term=Glavinic+U&cauthor_id=32948231)**^11^, Myrielle Dupont-Rouzeyrol^12^, Célestine M. Atyame^8^, Nicolas Pocquet^13^, Sébastien Boyer^14^, Catherine Dauga^15^, Marie Vazeille^15^, André Yébakima^16^, Michael T. White^17^, Constantianus J. M. Koenraadt^18^, Patrick Mavingui^8^, Anubis Vega-Rua^7^, Eva Veronesi^11^, Gorben P. Pijlman^9^, Christophe Paupy^10^, Núria Busquets^4^, Ricardo Lourenço-de-Oliveira^5^, Xavier De Lamballerie^19^, Anna-Bella Failloux^15*^**

^1^Institut Pasteur, Bioinformatics and Biostatistics Hub, F-75015 Paris, France

^2^Department of Vector Control, Center for Research, Diagnostic, and Reference, Institute of Tropical Medicine Pedro Kouri, PAHO-WHO Collaborating Center for Dengue and its Control, Havana, Cuba

^3^Institut Pasteur du Cambodge, Virology Unit, Cambodia

^4^IRTA, Centre de Recerca en Sanitat Animal (CReSA, IRTA-UAB), Campus de la Universitat Autònoma de Barcelona, 08193 Bellaterra, Spain

^5^Laboratorio de Mosquitos Transmissores de Hematozoarios, Instituto Oswaldo Cruz, Fiocruz, Rio de Janeiro, RJ, Brazil

^6^Centre for Research in Infectious Diseases, Department of Medical Entomology, Yaoundé, Cameroon

^7^Institut Pasteur of Guadeloupe, Laboratory of Vector Control research, Unit Transmission Reservoir and Pathogens Diversity, Les Abymes, Guadeloupe

^8^UMR PIMIT (Processus Infectieux en Milieu Insulaire Tropical), Université de La Réunion, INSERM 1187, CNRS 9192, IRD 249, Plateforme Technologique CYROI, Sainte-Clotilde, La Réunion

^9^Laboratory of Virology, Wageningen University, Wageningen, The Netherlands

^10^IRD, MIVEGEC, University of Montpellier, IRD, CNRS, Montpellier, France

^11^National Centre for Vector Entomology, Institute of Parasitology, Vetsuisse Faculty, University of Zürich, Zürich, Switzerland

^12^Institut Pasteur de Nouvelle-Calédonie, URE Dengue et Arboviroses, Nouméa, Nouvelle-Calédonie

^13^Institut Pasteur de Nouvelle-Calédonie, URE Entomologie Médicale, Nouméa, Nouvelle-Calédonie

^14^Institut Pasteur du Cambodge, Medical Entomology Unit, Cambodia

^15^Institut Pasteur, Arboviruses and Insect Vectors, F-75015 Paris, France

^16^VECCOTRA, Rivière Salée, Martinique

^17^Institut Pasteur, G5 Infectious Disease Epidemiology and Analytics, F-75015 Paris, France

^18^Laboratory of Entomology, Wageningen University & Research, Wageningen, the Netherlands

^19^Unité des Virus Emergents (UVE), Aix Marseille Université, IHU Méditerranée Infection, Marseille, France

*Corresponding author. Email: anna-bella.failloux@pasteur.fr

**Supplementary Table 1.** **Fifty mosquito populations included in this study.** The names of mosquito populations, country and locality of origin, year of collection, generation used, and number of mosquitoes tested are presented. A total of 8,138 mosquitoes were analyzed.

| **Species** | **Population name** | **Country** | **Locality** | **Collection date** | **Generation used** | **Number of mosquitoes tested** | **Reference** |
| --- | --- | --- | --- | --- | --- | --- | --- |
| *Aedes aegypti* | Trois bassins | La Reunion | Trois Bassins | 2014 | F27 | 192 | ^12^ |
|  | Cuiaba | Brazil | Cuiaba | 2018 | F2-F4 | 242 | ^14^ |
|  | Londrina | Brazil | Londrina | 2018 | F2-F4 | 236 |  |
|  | Manaus | Brazil | Manaus | 2018 | F2-F4 | 247 |  |
|  | Natal | Brazil | Natal | 2018 | F2-F4 | 203 |  |
|  | Rio de Janeiro | Brazil | Rio de Janeiro | 2018 | F2-F4 | 241 |  |
|  | Haiti | Haiti | Port-au-Prince | 2017 | F1 | 399 | ^38^ |
|  | Funchal | Madeira | Funchal | 2017 | F0 | 240 |  |
|  | Benoue | Cameroon | Benoue | 2017-2018 | F4 | 50 | ^24^ |
|  | Douala | Cameroon | Douala | 2017-2018 | F2 | 39 |  |
|  | Maroua | Cameroon | Maroua | 2017-2018 | F2 | 54 |  |
|  | Yaounde rural | Cameroon | Yaounde | 2017-2018 | F2 | 46 |  |
|  | Yaounde urban | Cameroon | Yaounde | 2017-2018 | F2 | 48 |  |
|  | Brazzaville | Congo | Brazzaville | 2017-2018 | F2 | 54 |  |
|  | Franceville | Gabon | Franceville | 2018 | F2 | 270 | ^50^ |
|  | LAUR | Guadeloupe | Point à Pitre | 2018 | F1 | 261 | ^11^ |
|  | St C | Guadeloupe | Saint-Claude | 2018 | F1 | 256 | unpublished |
|  | HAV-PT | Cuba | Pasteur | 2018 | F1 | 60 | ^51^ |
|  | HAV-PRG | Cuba | Parraga | 2018 | F1 | 62 |  |
|  | PP | Cambodia | Phnom Penh | 2019 | F1 | 360 | unpublished |
|  | Kone | New Caledonia | Koné | 2018 | F1 | 246 | ^14^ |
|  | Noumea | New Caledonia | Nouméa | 2018 | F1 | 195 |  |
|  | | | | | | |  |
| *Aedes albopictus* | Sainte-Marie | La Reunion | Sainte-Marie | 2019 | F0 | 192 | ^12^ |
|  | La Rivière | La Reunion | La Riviere | 2019 | F0 | 192 |  |
|  | Cuiaba | Brazil | Cuiaba | 2018 | F2-F4 | 229 | ^14^ |
|  | Londrina | Brazil | Londrina | 2018 | F2-F4 | 270 |  |
|  | Manaus | Brazil | Manaus | 2018 | F2-F4 | 204 |  |
|  | Natal | Brazil | Natal | 2018 | F2-F3 | 120 |  |
|  | Rio de Janeiro | Brazil | Rio de Janeiro | 2018 | F2-F4 | 270 |  |
|  | Yaounde urban | Cameroon | Yaounde | 2017-2018 | F2 | 24 | ^24^ |
|  | Tibati | Cameroon | Tibati | 2017-2018 | F2 | 24 |  |
|  | Douala | Cameroon | Douala | 2017-2018 | F2 | 43 |  |
|  | Brazzaville | Congo | Brazzaville | 2017-2018 | F5 | 24 |  |
|  | Mont | France | Montpellier | 2018 | F0 | 238 | ^52^ |
|  | Corse | France | Bastia | 2017 | F0 | 186 |  |
|  | Franceville | Gabon | Franceville | 2018 | F1 | 270 | ^50^ |
|  | Libreville | Gabon | Libreville | 2018 | F1 | 269 |  |
|  | Prat | Spain | El Prat de Llobregat | 2018 | F0-F1 | 93 | ^37^ |
|  | Rubí | Spain | Rubí | 2018 | F0-F1 | 157 |  |
|  | | | | | | |  |
| *Culex quinquefasciatus* | Sainte-Marie | La Reunion | Sainte-Marie | 2019 | F0 | 118 | ^12^ |
|  | Saint-Philippe | La Reunion | Saint-Philippe | 2019 | F1 | 60 |  |
|  | ABY | Guadeloupe | Pointe à Pitre | 2018 | F0 | 213 | ^11^ |
|  | ANB | Guadeloupe | Anse Bertrand | 2018 | F0 | 225 | unpublished |
|  | Dumbea | New Caledonia | Dumbea | 2018 | F0 | 238 | ^14^ |
|  | | | | | | |  |
| *Culex pipiens pipiens* | Cerdanyola del Vallès | Spain | Cerdanyola del Vallès | 2018 | F0 | 48 | unpublished |
|  | Best | The Netherlands | Best | 2014 | Lab colony | 133 | ^13^ |
| *Culex pipiens molestus* | Amsterdam | The Netherlands | Schiphol | 2013-2014 | Lab colony | 55 |  |
|  | | | | | | |  |
| *Aedes japonicus* | Steinbach | France | Steinbach | 2017 | F0 | 90 | ^40^ |
|  | Zurich | Switzerland | Zurich | 2017 | F0 | 90 |  |
|  | Lelystad | The Netherlands | Lelystad | 2017-2018 | F0 | 62 | ^44^ |

**Supplementary Table 2. Transmission efficiency (TE) in *Ae. aegypti* from Cameroon showing decreased ZIKV susceptibility around Benoué.** The *p*-values correspond to the comparison of each mosquito population against that from Benoué.

| **Species** | **Mosquito population** | **TE [95CI] (%)** | **P-value** |
| --- | --- | --- | --- |
| *Ae. aegypti* | Benoué | 34.4 [20.2–52.1] |  |
|  | Douala | 62.5 [42.2–79.2] | 0.0395 |
|  | Maroua | 33.3 [19–51.6] | 0.9309 |
|  | Yaoundé (rural) | 83.3 [63.1–93.6] | 0.0007 |
|  | Yaoundé (urban) | 79.2 [58.7–91.1] | 0.0015 |

**Supplementary Figure 1. Data structuration for *Aedes* mosquitoes studied in this experiment with respect to (A) mosquito sampling within countries and (B) usage of ZIKV strains to infect mosquitoes from a given country.** In all panels, available data is denoted by a blue tile. Panels present aggregated data over all possible values of days post-infection. In panel (A), the x-axis represents the country where mosquitoes were sampled and the y-axis the different locations within each country (1 or more locations). Panel (B) shows on the y-axis which ZIKV strain (prior to continent-level aggregation) was used to infect mosquitoes sampled in country from the x-axis.

**Supplementary Figure 2.** Standardized protocol of experimental infections of mosquitoes and parameters measured to assess vector competence for each combination mosquito population and ZIKV. The figure was built using Microsoft® PowerPoint®.


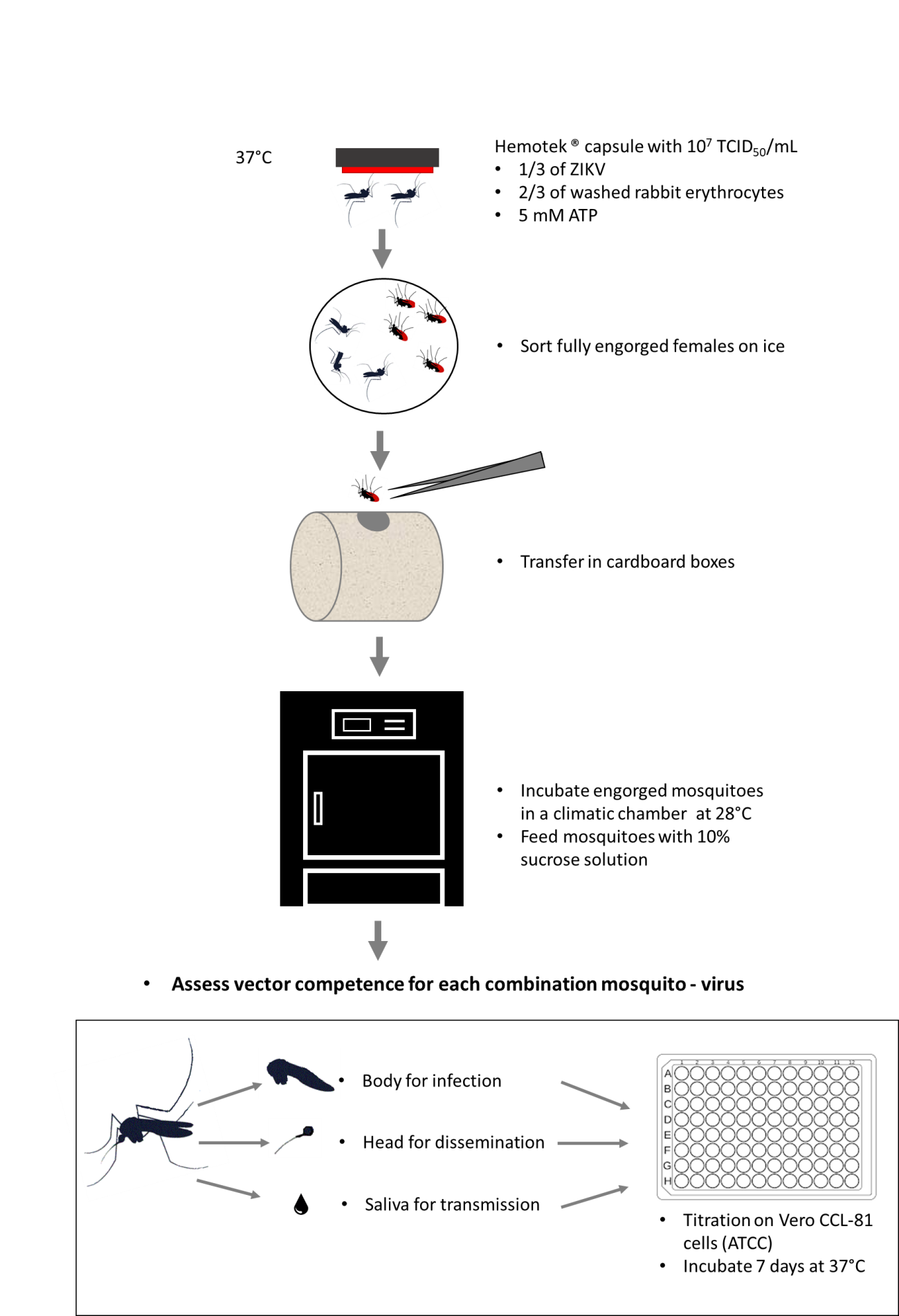


**Supplementary Figure 3. Test of different blood sources for optimizing infection rate (a) and dissemination efficiency (b).** One-week old *Aedes aegypti* PAEA mosquitoes (colony collected in French Polynesia in 1994; ^53^) were exposed to an infectious blood meal containing ZIKV Recife (KX197192; ^54^) provided at a titer of 10^7^ pfu/mL. After infection, engorged females were incubated 14 days at 28°C. Batches of mosquitoes were processed to estimate the infection rate (proportion of mosquitoes with infected body among tested mosquitoes) and dissemination efficiency (proportion of mosquitoes with infected head among tested mosquitoes). Three types of blood were tested: (i) rabbit (Institut Pasteur), (ii) rabbit (Charles River®), and (iii) bovine (Charles River®), denoted by the different colors. We showed that rabbit blood allowed optimal infection and dissemination of ZIKV. Each sample corresponds to two biological replicates (2 x 23-25 mosquitoes). Error bars represent the 95% confidence intervals. *p*-values are indicated for comparisons by two-sided Fisher’s exact test.


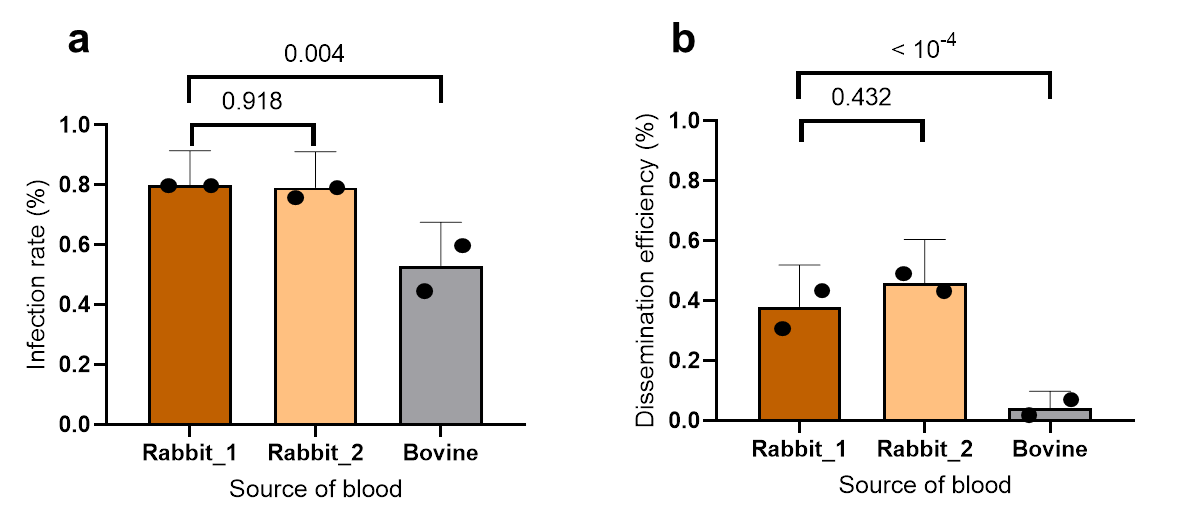


**Supplementary Figure 4.** **Maximum likelihood (PhyML) phylogenetic trees built using complete genomes of ZIKV, except for LR792671 (Guadeloupe) corresponding to partial ns5 sequence.** **(a)** Relative positions of the six ZIKV strains used for mosquito experimental infections. **(b)** Phylogenetic positions of the same strains among ZIKV from different geographic locations. Significant bootstrap values were displayed. See details on the six ZIKV strains studied in table below.


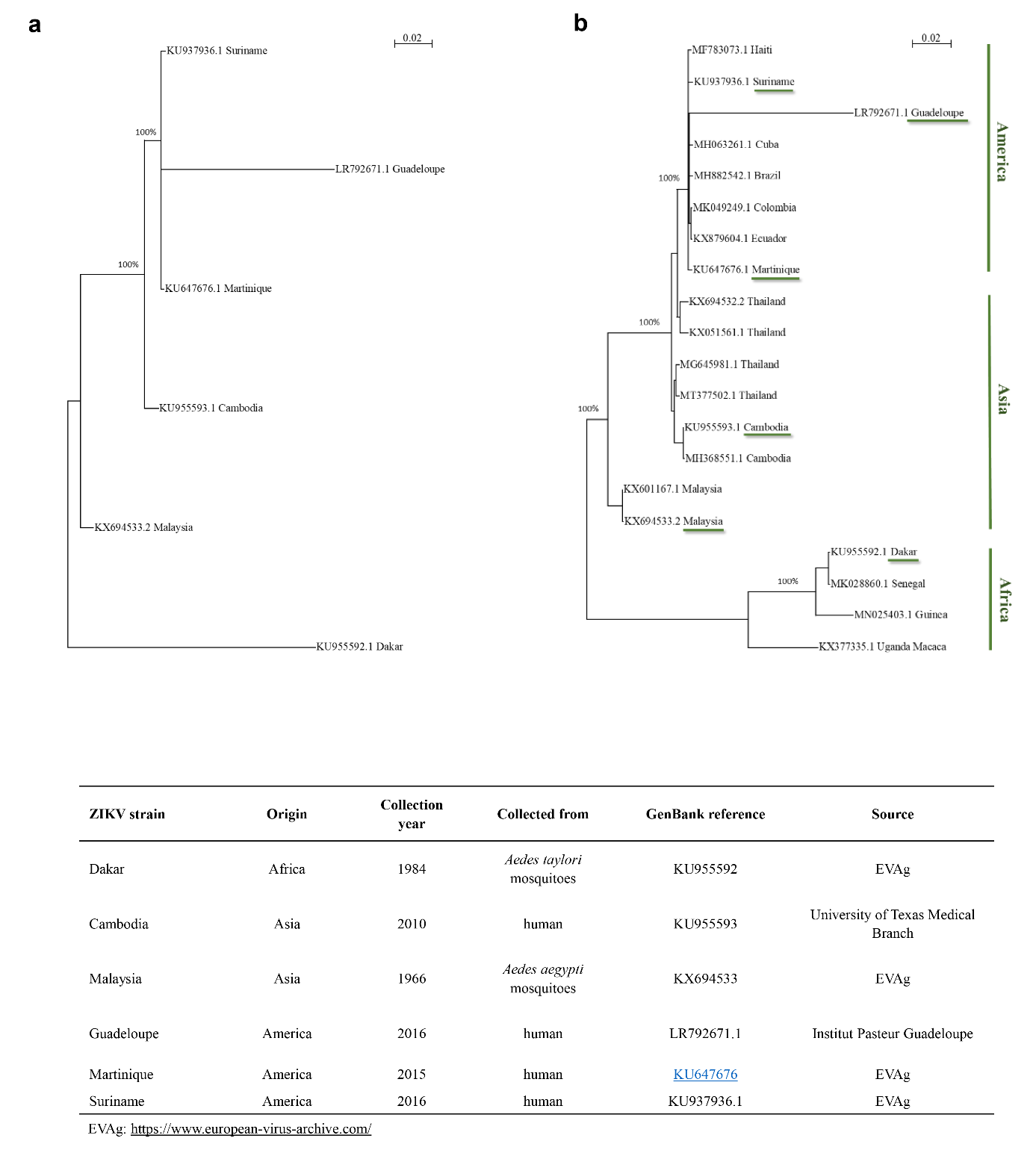


**Supplementary Figure 5. Model-predicted transmission efficiency of *Ae. aegypti* (A) and *Ae. albopictus* (B) mosquitoes according to time since infection, split by country of mosquito sampling.** Countries are ordered to reflect geographic proximity. Error bars show asymptotic 95% confidence interval from the mixed regression models centered on the average predicted TE. Raw coding of ZIKV strains was used instead of aggregating by continent. (n=30 biologically independent mosquitoes for each combination of species, country, mosquito population, days post-infection and ZIKV strain were studied, unless stated otherwise. Aggregation of ZIKV strains by continent result in n=150 mosquitoes per data point. See Table S1 for complete break-down of sample sizes). Colors correspond to ZIKV strains.

**Supplementary Figure 6. Transmission efficiencies of ZIKV strains at the regional level for *Ae. aegypti* and *Ae. albopictus* mosquitoes sampled at one or more locations in each studied country, at 21 dpi.** Vertical bars help identify different countries, shown on the x-axis with individual colors. Empty cells represent absence of data. Color gradients correspond to TE values (ranging 0–100%).

**Supplementary Figure 7. Model-predicted transmission efficiency of *Ae. aegypti* (A) and *Ae. albopictus* (B) mosquitoes according to time since infection, split by continent of mosquito sampling.** Countries are ordered to reflect geographic proximity. Error bars show asymptotic 95% confidence interval from the mixed regression models centered on the average predicted TE. Raw coding of ZIKV strains was used instead of aggregating by continent. (n=30 biologically independent mosquitoes for each combination of species, country, mosquito population, days post-infection and ZIKV strain were studied, unless stated otherwise. Aggregation of countries by continent result in varying sample sizes across panels, depending on number of countries. See Table S1 for complete break-down of sample sizes). Colors correspond to ZIKV strains.

**Supplementary Figure 8. Transmission efficiencies of ZIKV strains for *Ae. aegypti* and *Ae. albopictus* mosquitoes sampled at one or more locations in every studied country, at 21 dpi.** Regional variation within countries was discarded and countries regrouped by continent shown on the x-axis with individual colors and separated with vertical bars. Empty cells represent absence of data. Color gradients correspond to TE values (ranging 0–100%).

**Supplementary Figure 9. Model-predicted transmission efficiency of *Ae. aegypti* (A) and *Ae. albopictus* (B) mosquitoes according to time since infection, split by continent of mosquito sampling.** Countries are ordered to reflect geographic proximity. Error bars show asymptotic 95% confidence interval from the mixed regression models centered on the average predicted TE. ZIKV strains are aggregated by continent. (n=30 biologically independent mosquitoes for each combination of species, country, mosquito population, days post-infection and ZIKV strain were studied, unless stated otherwise. Aggregation of countries by continent result in varying sample sizes across panels, depending on number of countries. See Table S1 for complete break-down of sample sizes). Colors correspond to continent-agregated ZIKV strains.

**Supplementary Figure 10. Transmission efficiencies of ZIKV strains for *Ae. aegypti* and *Ae. albopictus* mosquitoes sampled at one or more locations in each studied country, at 21 dpi.** Regional variation within countries was discarded and countries regrouped by continent shown on the x-axis with individual colors and separated with vertical bars. ZIKV strains were pooled in a geographical clustering that reflected their phylogeny. Empty cells represent absence of data. Color gradients correspond to TE values (ranging 0–100%).
