# Supplementary material for: Zika vector competence data reveals risks of outbreaks: the contribution of the European ZIKAlliance project
Source: Nat Commun. 2022 Aug 2;13:4490. doi: 10.1038/s41467-022-32234-y (PMC9345287; doi:10.1038/s41467-022-32234-y)
Supplement: Supplementary file 2 — Reporting Summary [file 41467_2022_32234_MOESM2_ESM.pdf]

nature portfolio

Corresponding author(s): FAILLOUX Anna-Bella

Last updated by author(s): 2022-07-4

Reporting Summary

Nature Portfolio wishes to improve the reproducibility of the work that we publish. This form provides structure for consistency and transparency in reporting. For further information on Nature Portfolio policies, see our Editorial Policies and the Editorial Policy Checklist.

Statistics

For all statistical analyses, confirm that the following items are present in the figure legend, table legend, main text, or Methods section.

n/a	Confirmed	
☐	☒	The exact sample size (n) for each experimental group/condition, given as a discrete number and unit of measurement
☐	☒	A statement on whether measurements were taken from distinct samples or whether the same sample was measured repeatedly
☐	☒	The statistical test(s) used AND whether they are one- or two-sided<br>*Only common tests should be described solely by name; describe more complex techniques in the Methods section.*
☐	☒	A description of all covariates tested
☒	☐	A description of any assumptions or corrections, such as tests of normality and adjustment for multiple comparisons
☐	☒	A full description of the statistical parameters including central tendency (e.g. means) or other basic estimates (e.g. regression coefficient) AND variation (e.g. standard deviation) or associated estimates of uncertainty (e.g. confidence intervals)
☐	☒	For null hypothesis testing, the test statistic (e.g. F, t, r) with confidence intervals, effect sizes, degrees of freedom and P value noted<br>*Give P values as exact values whenever suitable.*
☒	☐	For Bayesian analysis, information on the choice of priors and Markov chain Monte Carlo settings
☒	☐	For hierarchical and complex designs, identification of the appropriate level for tests and full reporting of outcomes
☒	☐	Estimates of effect sizes (e.g. Cohen's d, Pearson's r), indicating how they were calculated

Our web collection on statistics for biologists contains articles on many of the points above.

Software and code

Policy information about availability of computer code

Data collection	Data were collected as Microsoft Excel spreadsheets and consolidated in the same software
Data analysis	Data analysis was performed using the R statistical software (v4.2.0) and the following packages: tidyverse (v1.3.1), lme4 (v1.1-27.1), emmeans (v1.7.2), partR2 (v0.9.1) and ggplot (v3.3.5)

For manuscripts utilizing custom algorithms or software that are central to the research but not yet described in published literature, software must be made available to editors and reviewers. We strongly encourage code deposition in a community repository (e.g. GitHub). See the Nature Portfolio guidelines for submitting code & software for further information.

Data

Policy information about availability of data

All manuscripts must include a data availability statement. This statement should provide the following information, where applicable:

- Accession codes, unique identifiers, or web links for publicly available datasets
- A description of any restrictions on data availability
- For clinical datasets or third party data, please ensure that the statement adheres to our policy

The data that support the findings of this study are fully available in an electronic supplementary table (Microsoft Excel XLSX format) at 10.5281/zenodo.6788898 as well as in the GitLab repository found in the Code availability section.

Human research participants

Policy information about studies involving human research participants and Sex and Gender in Research.

Reporting on sex and gender	Not applicable
Population characteristics	Not applicable
Recruitment	Not applicable
Ethics oversight	Not applicable

Note that full information on the approval of the study protocol must also be provided in the manuscript.

Field-specific reporting

Please select the one below that is the best fit for your research. If you are not sure, read the appropriate sections before making your selection.

- [x] Life sciences
- [ ] Behavioural & social sciences
- [ ] Ecological, evolutionary & environmental sciences

For a reference copy of the document with all sections, see nature.com/documents/nr-reporting-summary-flat.pdf

Life sciences study design

All studies must disclose on these points even when the disclosure is negative.

Sample size	No sample size calculation was performed as this study was designed to explore the interactions between ZIKV strains and mosquito populations from different geographical origins. Sample size was maintained identical across categorical strata.
Data exclusions	N = 90 mosquitoes (Cambodia), dissected at 10 days post-infection, were discarded from the analysis. This DPI value was only available for one country (Cambodia, having only one mosquito species (Ae. aegypti) challenged with 3 ZIKV strains) and could not be compared to any other available data we generated.
Replication	We used field-collected mosquitoes which do not feed very well on a blood source in BSL3 conditions. This number was low enough to allow for a single experiment to be conducted for each time point resulting in more than 30 mosquitoes available (target sample size for each data point).
Randomization	Mosquitoes of a same cage were randomly sorted to form groups of 50-60 female adults. There was no covariate involved in breeding of mosquitoes as cages were subject to comparable light and humidity conditions. Each of these batches was used for a single experimental infection. After infection, fully-fed mosquitoes were maintained in cardboard boxes until examination. At each time point (7, 14 and 21 days post-infection), a subset of N = 30 mosquitoes were sampled among the surviving ones. All these statements are included in the manuscript.
Blinding	Not applicable. There was no blinding as there was no covariate to blind experimenters or mosquitoes from.

Reporting for specific materials, systems and methods

We require information from authors about some types of materials, experimental systems and methods used in many studies. Here, indicate whether each material, system or method listed is relevant to your study. If you are not sure if a list item applies to your research, read the appropriate section before selecting a response.

Materials & experimental systems

n/a	Involved in the study	
[x]	[ ]	Antibodies
[ ]	[x]	Eukaryotic cell lines
[x]	[ ]	Palaeontology and archaeology
[ ]	[x]	Animals and other organisms
[x]	[ ]	Clinical data
[x]	[ ]	Dual use research of concern

Methods

n/a	Involved in the study	
[x]	[ ]	ChIP-seq
[x]	[ ]	Flow cytometry
[x]	[ ]	MRI-based neuroimaging

Eukaryotic cell lines

Policy information about cell lines and Sex and Gender in Research

Cell line source(s)	Vero CCL-81 (ATCC)<br>BHK-21 (ATCC)
Authentication	purchased from the ATCC company
Mycoplasma contamination	Vero CCL-81 cells and BHK-21 tested negative for mycoplasma contamination
Commonly misidentified lines<br>(See ICLAC register)	No commonly misidentified cell lines were used in this study.

Animals and other research organisms

Policy information about studies involving animals; ARRIVE guidelines recommended for reporting animal research, and Sex and Gender in Research

Laboratory animals	We used rabbits (Oryctolagus cuniculus), Charles River, New Zealand White male (2.7 - 3 kg) for blood to run experimental infections of mosquitoes.
Wild animals	The study did not involve wild animals.
Reporting on sex	*Indicate if findings apply to only one sex; describe whether sex was considered in study design, methods used for assigning sex. Provide data disaggregated for sex where this information has been collected in the source data as appropriate; provide overall numbers in this Reporting Summary. Please state if this information has not been collected. Report sex-based analyses where performed, justify reasons for lack of sex-based analysis.*
Field-collected samples	Mosquitoes were collected on the field as adults, larvae or eggs. Larvae were reared in pans supplemented with yeast tablets. Pupae were collected in containcers and placed in cages. Obtained adults were fed with 10% sucrose solution and maintained in chambers under controlled conditions (28°C, 70% relative humidity, 16:8 hour (Light:Dark) photoperiod).
Ethics oversight	All experiments using animals were approved by the Ethics committee of each country.

Note that full information on the approval of the study protocol must also be provided in the manuscript.
